# Development of Broadband High-Frequency Piezoelectric Micromachined Ultrasonic Transducer Array

**DOI:** 10.3390/s21051823

**Published:** 2021-03-05

**Authors:** Xu-Bo Wang, Le-Ming He, You-Cao Ma, Wen-Juan Liu, Wei-Jiang Xu, Jun-Yan Ren, Antoine Riaud, Jia Zhou

**Affiliations:** 1 State Key Laboratory of ASIC and System, School of Microelectronics, Fudan University, Shanghai 201203, China; xbwang16@fudan.edu.cn (X.-B.W.); arthur.lmhe@gmail.com (L.-M.H.); ycma19@fudan.edu.cn (Y.-C.M.); 14110720053@fudan.edu.cn (W.-J.L.); jyren@fudan.edu.cn (J.-Y.R.); antoine_riaud@fudan.edu.cn (A.R.); 2 Univ. Polytechnique Hauts-de-France, CNRS, Univ. Lille, Centrale Lille, UMR 8520-IEMN, DOAE, F-59313 Valenciennes, France; wei-jiang.xu@uphf.fr; 3 INSA Hauts-de-France, Le Mont Houy, F-59313 Valenciennes, France

**Keywords:** high-frequency, wide bandwidth, PMUT, PZT, PDMS, micromachining, two-dimensional array, rangefinder

## Abstract

Piezoelectric micromachined ultrasonic transducers (PMUT) are promising elements to fabricate a two-dimensional (2D) array with a pitch small enough (approximately half wavelength) to form and receive arbitrary acoustic beams for medical imaging. However, PMUT arrays have so far failed to combine the wide, high-frequency bandwidth needed to achieve a high axial resolution. In this paper, a polydimethylsiloxane (PDMS) backing structure is introduced into the PMUTs to improve the device bandwidth while keeping a sub-wavelength (λ) pitch. We implement this backing on a 16 × 8 array with 75 µm pitch (3λ/4) with a 15 MHz working frequency. Adding the backing nearly doubles the bandwidth to 92% (−6 dB) and has little influence on the impulse response sensitivity. By widening the transducer bandwidth, this backing may enable using PMUT ultrasonic arrays for high-resolution 3D imaging.

## 1. Introduction

Two-dimensional (2D) phased arrays are foreseen to revolutionize medical imaging with high frame rate, high scanning accuracy, and dynamic focusing [1]. Compared with bulk ultrasonic transducers, the micro-machined ultrasonic transducers (MUTs) are easy to fabricate even for high acoustic frequencies (over 10 MHz) [2], can be produced in large arrays with a small footprint [3], and mostly have high process compatibility with standard integrated circuit production [4]. Therefore, MUTs are promising elements for high-frequency 2D phased arrays. MUTs are divided into capacitive-based transducers (CMUTs) and piezoelectric-based transducers (PMUTs). Compared to CMUT, PMUTs have a more simple structure and thus a robust fabrication process [5], generate higher acoustic beam intensity, and do not require any bias voltage, which is preferred in medical applications [6]. However, the bandwidth of PMUTs is very limited and precludes the device from most imaging applications, including pulse-echo imaging and those involving chirp waveforms or harmonic response applications [7].

Several approaches exist to overcome this limitation. In mode-merging [8], a device with an appropriate structure design, such as a ring [7] or ribbon [9] shape, can generate two or more resonance peaks and overlap in one broad bandwidth, often exceeding 100% (−6 dB). However, such devices are challenging to design for high frequency (because the resonance peaks become relatively narrower) and the overlapping effect is sensitive to the mass density of the load [7]. Another approach better suited for high-frequency applications is using parallel multiple resonator cells with different working frequencies to form an effective element [10]. The independent resonance peaks overlap into one under load and get a large bandwidth at high frequency [11]. However, there exist two drawbacks in its further imaging applications: (i) an acoustic lens is needed to focus the beam produced by all the parallel cells [12], and (ii) the juxtaposition of many elements is hardly compatible with the pitch requirement of a 2D phased array, which limits the steering range. In summary, the existing technology cannot be applied to get a high-frequency broad bandwidth 2D PMUTs phased array.

In bulk devices, the bandwidth is increased without changing the element size by adding a backing layer. Such a layer requires high attenuation and good acoustic matching with the vibrating element [13]. To the best of our knowledge, this backing solution has never been applied to high-frequency PMUTs. This may be down to the specific requirements of PMUTs compared to bulk devices. First, the backing layer fabrication process must be compatible with microfabrication techniques. Second, because PMUTs rely on a flexural vibration mode and have an acoustic impedance close to water or human tissues, the backing impedance must also match that of water (unlike PZT for bulk elements).

PDMS is selected as the backing material for the following reasons. Firstly, PDMS has low surface energy and easily fills delicate structures under vacuum [14]. Secondly, the relatively soft PDMS works as an extra load without changing the plate flexural vibration mode to the thickness mode as bulk devices do. Thirdly, the acoustic properties of PDMS, including attenuation, are well characterized, and the properties can be potentially changed by modifying composition or adding particles, such as tungsten microspheres [15], as required.

In this paper, we demonstrate a backed PMUTs (B­PMUTs) with a pitch of 3λ/4. The backing layer is obtained by deep silicon etching and PDMS backfilling into the etched blind hole (cavity). After describing the device fabrication and structure, we measure the resulting vibration mode of the B-PMUT, the equivalent damping effect and attenuation of the PDMS backing structure, and the pulse response sensitivity of the B-PMUT. Finally, the 2D array is compared to the existing literature in terms of pitch, frequency, and bandwidth.

## 2. Materials and Methods

### 2.1. Design and Fabrication

**Figure 1:**
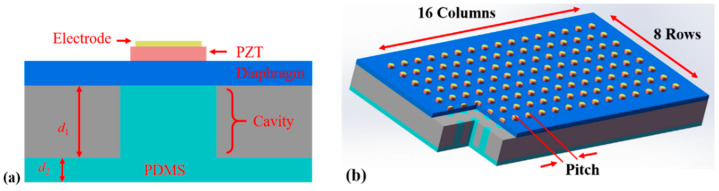
Schematic of backed piezoelectric micromachined ultrasonic transducers (B-PMUTs). (**a**) The designed structure of a single element. (**b**) Three-dimensional (3D) schematic of the array.

The structure of the device is shown in Figure 1a, and the multilayered structure is composed of, from top to bottom, a top electrode, a lead-zirconate-titanate (PZT) piezoelectric layer, a vibration diaphragm (including bottom electrode, insulating silicon oxide film, single crystal Si, and buried silicon oxide layer), cavity structure, and PDMS backing. The array consists of 16 × 8 elements with a 75 µm pitch (3λ/4, at 15 MHz in water), as shown in Figure 1b [16]. The corresponding structure parameters are listed in Table 1.

**Figure 2:**
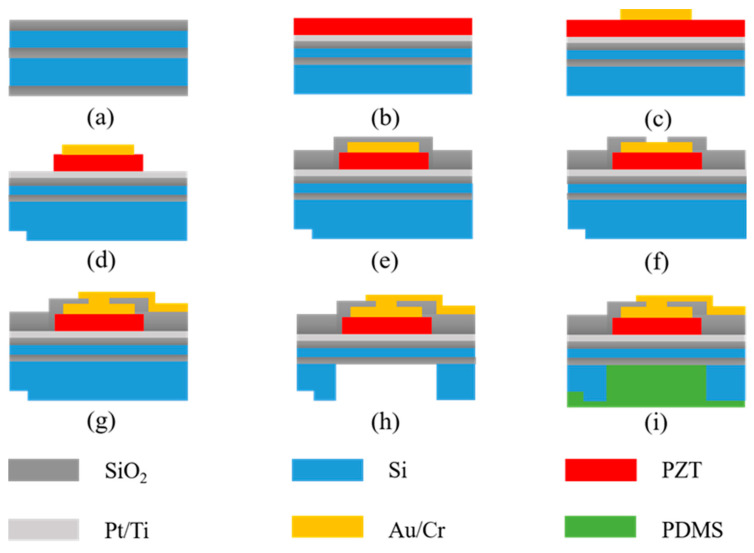
(**a**–**i**) The fabrication process of the B-PMUTs.

The fabrication process is shown in the following panels in Figure 2. (a) A four inches silicon-on-insulator (SOI) wafer with a 4 μm device layer (single crystal Si) and a 500 nm buried oxide layer is wet oxidized at 1100 °C to get 500 nm thermal oxide layers on both sides. (b) The wafer is back-grinded to 300 μm, and then 20 nm/200 nm of Ti/Pt is deposited by physical vapor deposition (PVD) as the bottom electrode, after which a 1 μm PZT film is spin-coated by a multilayer sol-gel process. The PZT thin film deposition is finished by a commercial company (ZILM Co., http://www.zilm-tech.com/, accessed on 7 February 2021). (c) The 10 nm/100 nm Cr/Au top electrode is deposited by PVD and patterned via the lift-off process. (d) The PZT film is patterned via wet etching with positive photoresist (RZJ-304) as a mask. By exposing the wafer to a 20:6:1 mixture of deionized water /hydrochloric acid/buffered oxide etchant (BOE, 6:1) with stirring, and immediately followed by a vigorous rinsing in DI water, cylinder-like islands are obtained with 40 s etch for 1 μm PZT at room temperature. (e) A 250 nm silicon oxide layer is deposited at 300 °C (below the curie point of PZT) to be the insulation and passivation layer using plasma-enhanced chemical vapor deposition (PECVD). (f) For wire contact, a via hole is etched using reactive ion etching (RIE). (g) A metal stack of 20 nm chromium and 200 nm gold is patterned by lift-off to be the lead wires and pads. (h) The device layer is released by deep reactive ion etching (DRIE) from the backside of the wafer, and a cavity of depth *d*_1_ = 300 µm is remaining. (i) The backing is formed by pouring a 10:1 polydimethylsiloxane (PDMS) mixture (Sylgard 184, Dow Corning Co., Midland, TX, USA) into the opening end of the cavity and placing the chip in a vacuum (below 133 Pa) to let the silicone spontaneously fill the cavity. Most of the excess PDMS is eliminated by spin-coating, and the remaining forms a thin film of thickness *d*_2_ = 30 µm. After curing, the PDMS fits tightly with the PMUT diaphragm and forms a thin layer over the whole SOI substrate.

**Figure 3:**
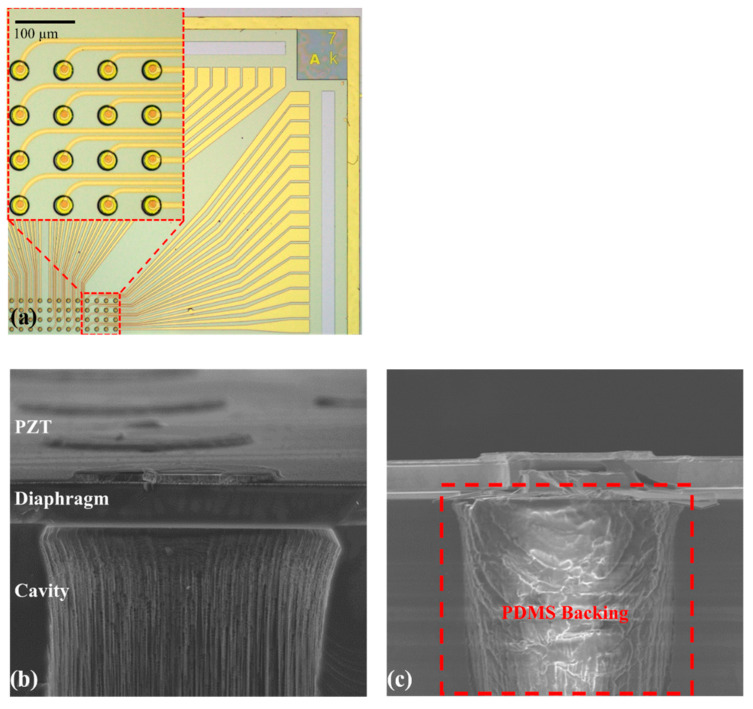
Microscopy pictures of B-PMUTs. (**a**) Top view of a part of 16 × 8 PMUTs arrays. (**b**) Cross-sectional SEM image of a typical element without PDMS backing. (**c**) Cross-sectional SEM image of the element with PDMS filling.

The chip is zoomed-in and shown in Figure 3a; the array consists of 16 × 8 addressable elements, and each element can be driven independently for future phase control. Figure 3b,c show the SEM cross-section of the typical element without and with PDMS filling, respectively. The PZT layer and Si diaphragm on an SOI substrate with a deeply etched cavity are visible, and the diameter of the cavity is 40 µm.

### 2.2. Experimental Setup

To verify the performance of the PDMS backing, the devices of B-PMUTs are compared to a control group without PDMS backing (C-PMUTs). The two groups of devices are fabricated in the same batch; i.e., the only difference between them is with and without the backing layer.

**Figure 4:**
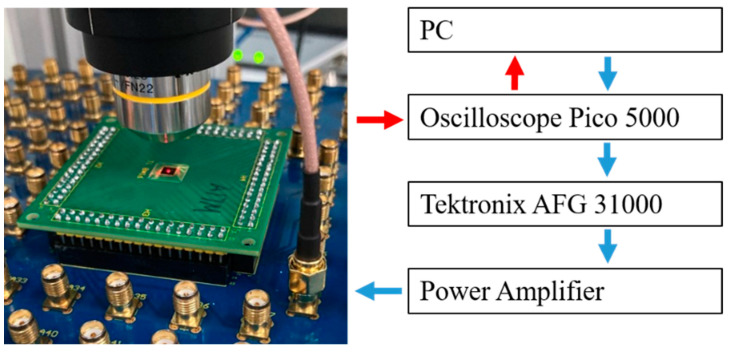
Laser Doppler vibrometer (LDV) setup for characterizing the PMUT performance.

We first analyze the effect of PDMS on the dissipation and loading of the PMUT membrane based on the steady-state responses of the B- and C-PMUTs in air and then in water. In this experiment, a 4 V_pp_ 100-cycles sine wave is used as the excitation signal. Then, to verify that the thickness of PDMS is enough and suitable to absorb the rear reflection, we measure the effect of dissipation of the baking layer on the pulse responses of the B-PMUTs in air (to eliminate the additional loading due to water that may enhance the bandwidth). In this experiment, the devices are tested with a 10 ns-long 30 V pulse, which is generated by an arbitrary function generator (Tektronix AFG3100) and an amplifier (Mini-circuits ZHL-5W-1+). The vibration of the diaphragm in the experiment is measured by a scanning heterodyne laser Doppler vibrometer (LDV) [17] and analyzed in the Python environment. The experimental setup is shown in Figure 4. Finally, we quantify the improvement in pulse sensitivity and bandwidth in a more realistic setting by comparing the pulse response in water with and without backing. The test method is in accordance with that in the dissipation measurement.

## 3. Results and Discussion

### 3.1. Steady-State Response

In air, the resonant frequency of the C-PMUT is 20.2 MHz with a displacement amplitude of 36.6 nm at the center of the tested element. In the same condition, the resonant frequency of the B-PMUT is 19.0 MHz with an amplitude of 9.2 nm.

**Figure 5:**
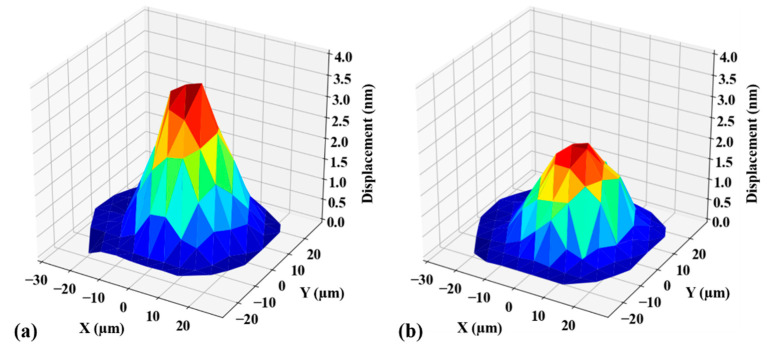
The first resonance mode of the devices working under water load for (**a**) a control group without PDMS backing (C-PMUT) and (**b**) B-PMUT.

The vibration results of the two group devices working under water load are shown in Figure 5. It can be seen that the two group devices are working in the same flexural vibration mode. Due to water loading, the resonant frequency of the C-PMUT is reduced to 16.4 MHz with the peak amplitude decreasing to 3.7 nm, which is about 10% of that in air. Similarly to the C-PMUT, the resonance frequency of the B-PMUT decreases to 15.3 MHz with the peak amplitude down to 1.9 nm, which is about 20% of that in air. The difference between C-PMUT and B-PMUT is coming from the additional PDMS backing.

To analyze the role of backing in the vibration process, the vibration of C-PMUT and B-PMUT in water load is fitted with an equivalent point-mass model in the form of $m\ddot{Z}+b\dot{Z}+kZ=F_{0}e^{j\omega t}$, where *m*, *b*, *k*, *F*_0_ are the equivalent mass, damping, stiffness, and piezoelectric driving force, respectively [10]. We obtain the value of these parameters from static and steady-state experiments, with $k=F_{0}/A_{Q}$, $b=F_{0}/\left(A_{D}\omega_{0}\right)$, and $m=k/\omega_{0}^{2}$, where *A*_Q_ is static displacement (C-PMUT and B-PMUT are found experimentally to share the same *A*_Q_ ), *A*_D_ is steady-state displacement, and $\omega_{0}$ is resonance angular frequency. Numerically, the ratio of equivalent parameters of C-PMUT and B-PMUT in water load are $k_{C}/k_{B}=1$, $b_{C}/b_{B}=0.47$, and $m_{C}/m_{B}=0.87$. This indicates that after adding the PDMS backing, the equivalent stiffness of the system is kept constant, the equivalent mass is increased by 15%, and the equivalent damping is doubled. Thus, the additional PDMS is backing half the Q-factor of the system.

### 3.2. Attenuation of PDMS Backing

**Figure 6:**
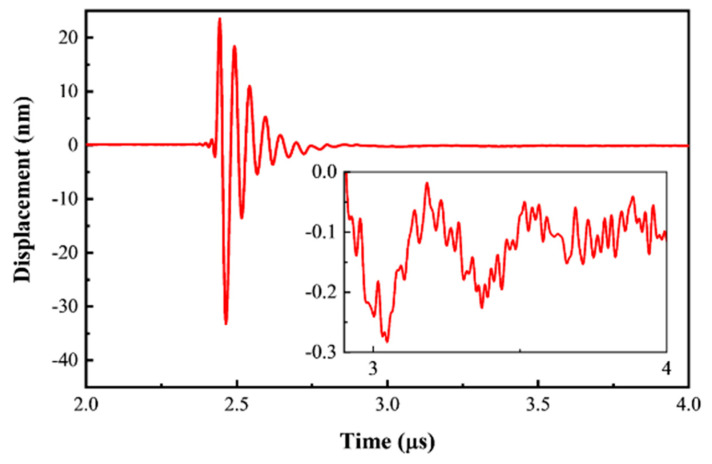
Peak displacement at the center of a B-PDMS element tested in air with a 10 ns 30 V pulse. The insert figure shows the detailed response from 2.9 to 4 µs.

The PDMS backing should be long enough to absorb the reflection wave from the rear face. Otherwise, it would disturb the measurement results of the impulse reponse. The attenuation effect of a 300 μm PDMS backing layer is tested in air, in which case the backing layer is the only way for reflection. As shown in Figure 6, the peak displacement amplitude is 33.2 nm at 2.46 µs, and no evident echoes are recorded after that.

There could be two reflections of the transmitted sound waves from the rear face. The first one occurs at the edge of the cavity, and the second one is 30 µm farther at the interface between the PDMS film and the air [18]. Considering the longitudinal velocity of PDMS (v) is about 1028 m/s [19], the two reflection disturbances should act on the vibration plate after 2*d*_1_/*v* = 0.58 µs and 2(*d*_1_ + *d*_2_)/*v* = 0.64 µs of the generation, respectively, which means that if they existed, both should appear after 3 µs. However, as shown in the insert of Figure 6, only a low-frequency wave (0.5 MHz) can be seen, which is possibly due to the thermal expansion of the PDMS [20]. No disturbance with a frequency higher than 5 MHz and peak-to-peak value higher than 0.1 nm is observed. Therefore, it can be deduced that the 300 µm PDMS backing structure can attenuate the reflected wave by at least 55 dB. This is much higher than the 7 dB attenuation of longitudinal waves in bulk [19], suggesting a complex propagation in the cylinder.

### 3.3. Impulse Response

**Figure 7:**
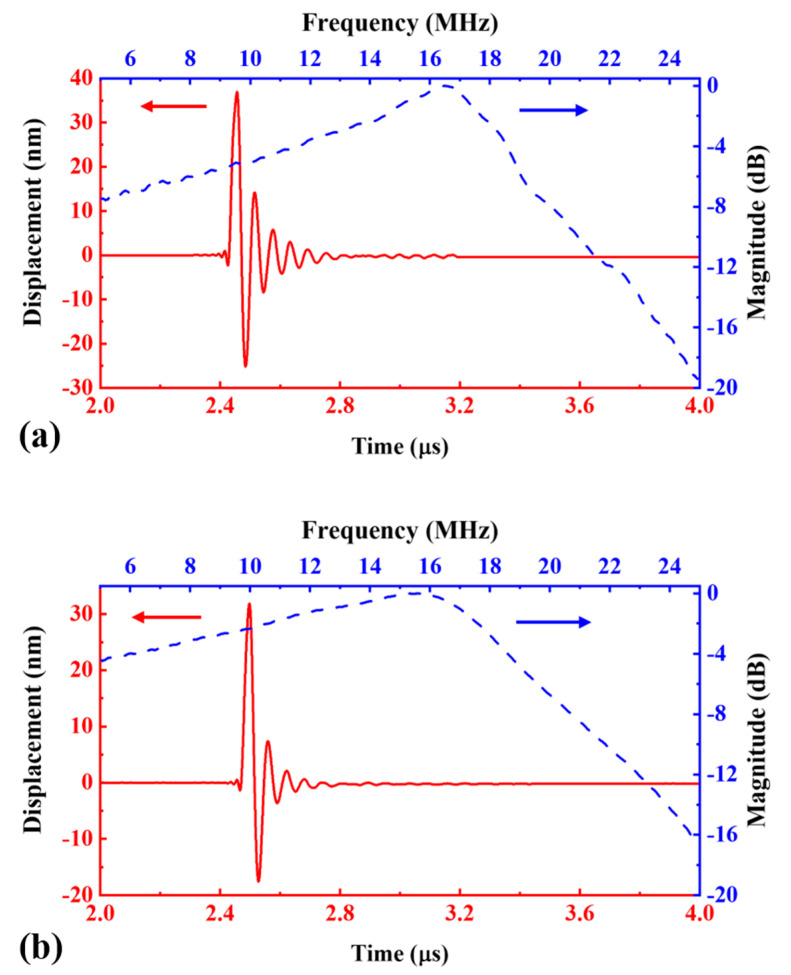
Vibration response of (**a**) C-PMUT and (**b**) B-PMUT, tested in water with a 10 ns 30 V pulse. The time-domain response is shown with a red solid line, and the frequency-domain response is shown with a blue dash line.

To analyzed the improvement of PDMS backing on the bandwidth, the impulse response of C-PMUT and B-PMUT are tested and compared. In Figure 7, the time-dependent displacement and the corresponding spectrum are shown in solid red line and dashed blue line, respectively. The experiment results are concluded in Table 2.

With water load, as shown in Figure 7a, the center frequency of C-PMUT is 16.5 MHz, and the peak amplitude is 36.9 nm, which is 94% of that in air, yielding a sensitivity of 1.2 nm/V. The decrease is not as much as that in the steady state because the peak amplitude of the impulse response is a synthesis of broadband response in transient. However, the fractional bandwidth increases sharply to 32% @−3 dB. This is because the water load increases the energy loss of the vibration system. The ringdown time is 160 ns @−20 dB. For the B-PMUT, as shown in Figure 7b, the center frequency is 15.6 MHz, which is 94.5% of that of C-PMUT. The peak amplitude is 31.8 nm, which is 86% of that of C-PMUT. The corresponding sensitivity is 1.1 nm/V. The above results indicate that the PDMS backing layer has little influence on the center frequency and pulse sensitivity. We think this is because the acoustic impedance of PDMS backing is matching with the impedance of water. The bandwidth of the B-PMUT is twice that of the control group, which is 63.4% @−3 dB (92% @−6 dB), and the ringdown time falls to 62 ns. This variation is favorable for medical imaging, and it is in accordance with the Q-factor predicted by the model given in Section 3.1.

**Figure 8:**
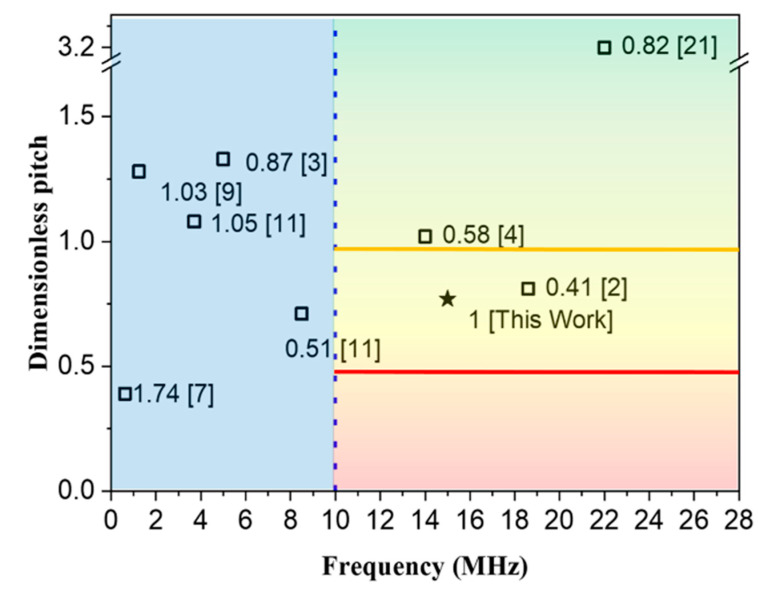
Comparison of working frequency, pitch, and bandwidth of different types of PMUTs under fluid load. The values in the label are the bandwidths (from references) normalized to our study. The hollow square represent the results in the published literature, and the solid star represents the results given in this study.

Figure 8 compares the characteristics of published PMUTs with the B-PMUT presented here for the potential of imaging. The figure is divided into low frequency (left) and high-frequency quadrants (right) according to the center frequency (10 MHz), and the right side is further developed into three quadrants depending on the dimensionless pitch (the ratio of pitch to the wavelength at a center frequency in the corresponding medium) [2,4,21]. The region between the yellow and red line is suitable for a 2D array, and the red line is the most ideal state for a phased array with an arbitrary steer angle [22]. Note that smaller pitches would not improve further the resolution for propagative waves. Compared with the devices in the right part, the B-PMUT moves closer to the red line, and while keeping a high bandwidth, nearly twice that of the counterpart [2], which promises higher axial resolution in imaging.

## 4. Conclusions

To overcome the bandwidth-pitch tradeoff of the 2D PMUTs array, we propose using a backing layer. We settle to use PDMS as the backing material because it easily fills small cavities, has an acoustic impedance close to water or human tissues, and has a strong damping effect without affecting the flexural vibration of the PMUT. After fabricating a 16 × 8 array with 75 µm pitch (3λ/4), we observe that the PDMS backing can double the bandwidth of the device with little influence on the center frequency and impulse response sensitivity. This technology is a step forward in developing a high-frequency PMUT phased array, which is expected to be widely used in superficial organ imaging, intravascular imaging, and other applications.

## Figures and Tables

**Table 1:** The structure parameters.

Name	Value (µm)	Name	Value (µm)
Electrode diameter	28	Cavity diameter	40
PZT diameter	32	Cavity depth (*d*_1_)	300
PZT thickness	1	PDMS thickness (*d*_1_ *+ d*_2_)	330
Diaphragm thickness	4.7	Pitch	75

**Table 2:** Impulse responses of C-PMUT and B-PMUT.

Device	Displacement (nm)		Ringdown Time (ns)		Center Frequency (MHz)		Bandwidth (@−3 dB)	
	Air	Water	Air	Water	Air	Water	Air	Water
C-PMUT	38.9	36.9	1710	160	20.2	16.5	6%	32%
B-PMUT	33.2	31.8	182	62	19.4	15.6	22%	63%

## Data Availability

Data sharing not applicable.
